# Multidomain Dementia Risk Reduction in Primary Care is Feasible: A Proof-of-concept study

**DOI:** 10.3233/JAD-240229

**Published:** 2024-06-11

**Authors:** Stephanie Van Asbroeck, Sebastian Köhler, Sophie C.P.M. Wimmers, Jean W.M. Muris, Martin P.J. van Boxtel, Kay Deckers

**Affiliations:** a Department of Psychiatry and Neuropsychology, Alzheimer Center Limburg, Mental Health and Neuroscience (MHeNs) Research Institute, Maastricht University, Maastricht, the Netherlands; b Department of Family Medicine, Careand Public Health Research Institute (CAPHRI), MaastrichtUniversity, Maastricht, the Netherlands

**Keywords:** Alzheimer’s disease, dementia, health behavior, lifestyle, prevention and control, primary health care, risk factors, telemedicine

## Abstract

**Background:**

Dementia risk reduction is a public health priority, but interventions that can be easily implemented in routine care are scarce.

**Objective:**

To evaluate the feasibility of integrating dementia risk reduction in regular consultations in primary care and the added value of a dedicated smartphone app (‘MyBraincoach’).

**Methods:**

188 participants (40–60 years), with modifiable dementia risk factors were included from ten Dutch general practices in a cluster-randomized trial (NL9773, 06/10/2021). Practices were randomly allocated (1 : 1) to provide a risk-reduction consultation only or to additionally provide the app. During the consultation, participants learned about dementia risk reduction and how to improve their risk profile. The app group received daily microteaching-notifications about their personally relevant risk factors. Feasibility was evaluated after 3 months using questionnaires assessing knowledge on dementia risk reduction and health behavior change. The primary outcome was change in the validated “LIfestyle for BRAin health” (LIBRA) score. In-depth interviews were conducted with participants and primary care providers (PCPs).

**Results:**

The interventions were positively perceived, with 72.0% finding the consultation informative and 69.2% considering the app useful. Drop-out was low (6.9%). LIBRA improved similarly in both groups, as did Mediterranean diet adherence and body mass index. Knowledge of dementia risk reduction increased, but more in the app group. Interviews provided insight in participants’ and PCPs’ needs and wishes.

**Conclusions:**

Integrating dementia risk reduction in primary care, supported by a smartphone app, is a viable approach towards dementia risk reduction. Larger trials are needed to establish (cost-)effectiveness.

## INTRODUCTION

Due to the identification of modifiable risk and protective factors for Alzheimer’s disease and related dementias, dementia risk reduction has become a major avenue for attenuating the rising prevalence of dementia and associated burden of disease [1–4]. Potentially modifiable risk factors for dementia include obesity, hypertension, hearing impairment, dyslipidemia, type 2 diabetes, depression, smoking, and physical inactivity. Protective factors include the adherence to a Mediterranean diet, and high cognitive and social activity engagement [4, 5]. In 2015, the Finnish Geriatric Intervention Study to Prevent Cognitive Impairment and Disability (FINGER) was the first randomized controlled trial (RCT) to demonstrate small but positive effects of a multidomain lifestyle intervention on change in cognitive performance [6]. Since then, the World-Wide FINGERS Network is aiming to provide further causal evidence from similar lifestyle interventions that vary in form, intensity, and target population [7]. These trials are essential for evaluating the efficacy of lifestyle interventions, but are costly, logistically demanding, and labor intensive, and often use restrictive inclusion criteria. Hence, they are less suited for population-wide implementation of brain health promotion [8–10].

Integrating dementia risk reduction in a primary care setting has been proposed as a potentially advantageous and feasible approach [11–13]. Firstly, primary care providers (PCPs) reach many individuals and could therefore play a key role in conveying this health message. In the Netherlands, all citizens are registered with a PCP who acts as a gatekeeper to specialized medical care. Secondly, most modifiable risk factors for dementia overlap with those for cardiometabolic health conditions and may already be routinely checked and managed long-term within primary care, as is the case in the Netherlands (e.g., via advice on lifestyle changes, antihypertensive or lipid-lowering medication). Moreover, PCPs can refer individuals to other healthcare professionals or organizations for additional or more specialized care or support. Importantly, discussing brain health at the general practice could lead to the initial awareness that lifestyle factors can influence brain health, which may act as an additional stimulus for behavior change [11, 14, 15].

Therefore, a proof-of-concept cluster-randomized trial named PRIMary care App-supported Brain health promotion (PRIMA-Brain) was conducted to examine the feasibility of a three-month-long blended brain-health promotion intervention in primary care. Specific objectives were to (1) assess the acceptability of the intervention based on the number of participant withdrawals and the perspectives of participants and PCPs on the intervention, (2) assess the demand for the intervention by exploring the perspectives of participants and PCPs hereon, and by assessing the usage of the brain-health promotion smartphone app, (3) explore the integration and possible future implementation of the intervention via interviews with PCPs, and (4) conduct a preliminary efficacy assessment based on changes in a validated comprehensive modifiable dementia risk score (i.e., “LIfestyle for BRAin health” score (LIBRA [5, 16])) during the intervention period, and changes in knowledge about dementia risk reduction [17].

## METHODS

### Design

**Fig. 1:**
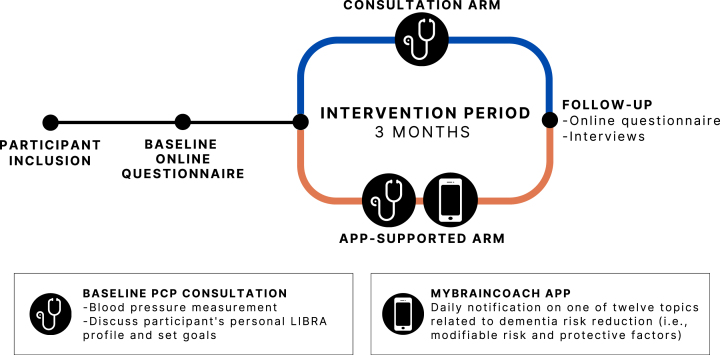
Participant journey. PCP, primary care provider; LIBRA, LIfestyle for BRAin health.

The PRIMA-Brain study took place between December 2021 and August 2023 in ten general practices located predominantly in the province of Limburg, the Netherlands. PCPs/general practices were recruited via a personalized invitation by e-mail (including several reminders (by phone)), which were sent to more than 20 practices with an interest in scientific research within the network of the research team and the Department of Family Medicine of Maastricht University. Next, the research team contacted the general practice to schedule an appointment to discuss participation. Additionally, a dedicated call for participation was published in the regular magazine of the Department of Family Medicine of Maastricht University. All practices were randomized in a 1 : 1 ratio to one of two intervention arms: (a) dementia risk reduction consultation with the PCP or (b) consultation+provision of a smartphone app to promote a brain-healthy lifestyle. Based on power analysis (effect size = 0.6 (difference in total LIBRA score by one altered LIBRA factor); standard deviation = 1; two-sided testing; α = 0.05; power = 0.90) and anticipating a 33.3% dropout rate, 180 participants needed to be included, i.e., an average of 18 patients per practice. After an online baseline questionnaire and the baseline study visit with the participant’s PCP, the intervention lasted approximately three months and ended with an online follow-up assessment. The complete participant journey is visualized in Fig. 1. In addition, interviews were conducted with a subgroup of participants in the app-supported intervention arm, as well as with PCPs. The study was approved by the medical ethical committee of Maastricht University Medical Center+(approval code: METC 20-080). Participants (voucher of 20.00 EUR/22.00 US dollars) as well as participating general practices received a small financial compensation (1,000.00 EUR/1,086.00 US dollars per practice). All participants provided their written informed consent. The study is registered in the Dutch National Trial Register (since October 6, 2021, NTR: NL9773).

### Study population

Participants were registered patients from ten general practices in the South of the Netherlands. Inclusion criteria were the following: 40–60 years old; having at least one modifiable risk factor for dementia (based on LIBRA) [5, 16]; being proficient in the Dutch language; and owning a smartphone with internet access. Exclusion criteria were the following: active episode of major depression, which made the individual unsuitable to participate according to the PCP; a dementia diagnosis; medical conditions because of which (app-)suggested lifestyle changes could not be made by default (e.g., certain movement constraints); and having previously used the “MyBraincoach” app. Patients eligible based on age (40–60 years) were informed via a letter, e-mail, or by phone about the possibility to participate. If the patient was interested to participate, they received a full participant information letter after which informed consent was obtained and full eligibility was confirmed.

### Intervention

**Fig. 2:**
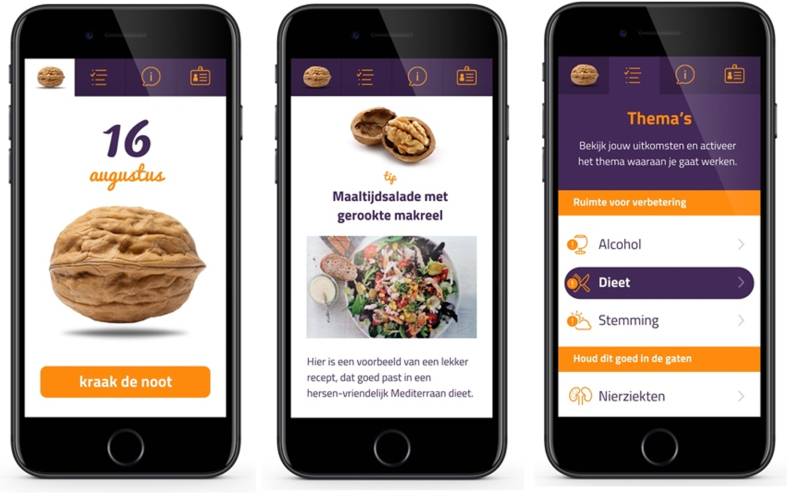
Screenshots of the MyBraincoach app.

At baseline, all participants had a consultation with their PCP, during which their dementia risk reduction profile (i.e., LIBRA profile) was discussed. An example of this profile is included in Supplementary Material 1. Personal room for improvement was discussed and individual, feasible lifestyle goal(s) were set. Participants in the app-supported arm additionally received access to a brain health promotion smartphone app (i.e., ‘MyBraincoach’, or ‘MijnBreincoach’ in Dutch [18]). This smartphone app shows users their modifiable dementia risk profile, consisting of the same 12 LIBRA risk and protective factors, after which they can activate a topic to work on. Users then receive a daily notification to “crack a nut” on their chosen topic (e.g., physical activity, healthy diet) including educational information, advice/tips, challenges, or quiz items, and framed in a positive and motivating manner. Per topic, 14 daily “nutifications” are sent over a period of two weeks (if the user opens a notification every day). Participants were free to change the active topic whenever they liked. Figure 2 shows a selection of app screenshots. Participants in the consultation-only arm were not provided with the app. No written materials were provided to both arms.

### Measures

Participants completed an online survey before (baseline) and after (three months follow-up) the intervention via the Castor Electronic Data Capture system [19]. The complete survey can be found in Supplementary Material 2. The surveys covered demographics, lifestyle factors, and health conditions included in LIBRA, and knowledge on dementia risk reduction. Self-reported health conditions were complemented by the participants’ health record data.

#### Demographics

Sex, age, marital status, and level of education were surveyed. The participants’ area-based neighborhood socioeconomic status (SES) was determined based on their four-digit postal code. Every postal code in the Netherlands has an assigned measure of SES, which is determined based on the financial welfare, educational level, and recent employment history of all private households within this postal code. This measure was obtained from Statistics Netherlands (25). Neighborhoods with a score < –0.1 were categorized as low SES, whereas neighborhoods with a score > 0.1 were categorized as high SES. Neighborhoods with scores between –0.1 and 0.1 were categorized as middle SES [20].

#### Lifestyle factors and health conditions

LIBRA is a comprehensive modifiable dementia risk score summarizing the presence or absence of 12 modifiable risk and protective factors into one numeric value and has been extensively validated to predict cognitive decline, incident dementia, and biomarkers of brain damage [21–26]. Here, the total LIBRA score and underlying individual risk and protective factors were used as outcome measures [27]. Lifestyle factors included in LIBRA were assessed at baseline and follow-up using questionnaires. Items on smoking behavior, alcohol consumption, body height and weight (to calculate body mass index (BMI)) were included [28]. Low-to-moderate alcohol consumption was defined as adherence to the Dutch guidelines of maximum seven standard units of alcohol per week [29]. Obesity was defined as a BMI≥30 kg/m^2^. Physical activity level was assessed using the European Prospective Investigation into Cancer and Nutrition (EPIC) physical activity questionnaire [30]. Participants were then considered physically inactive if they were inactive or moderately inactive based on the Cambridge Physical Activity Index [31]. Lifetime engagement in cognitively stimulating activities was measured via the Cognitive Reserve Index questionnaire (CRIq) at baseline [32]. This scale consists of three subscales: education, working activity, and leisure time. Participants answer since when and how frequent they have been engaged in specific activities during their life, and answers result in a total score commonly ranging from about 70 to 160 [32]. A CRIq score of ≥130 was considered cognitively active. At follow-up, custom items were used that asked about the current frequency of engagement in activities included in the leisure time CRIq subscale, relative to the frequency at baseline. These custom items were used to detect potential changes to cognitive activity engagement over the three-month study period, which is not possible with the original CRIq. Changes to education or working activity over three months were considered unlikely in our study population and thus not included at follow-up. These custom items were summed for every individual and standardized into z-scores. Individuals with a z-score below or equal to –1 (i.e., a one standard deviation (SD) or worse change in leisure time activity frequency than the population mean) got categorized as “low cognitively active” at follow-up. Individuals with a value above or equal to 1 (i.e., a one SD or better change in leisure time activity frequency than the population mean) got categorized as “high cognitively active”. All others got allocated the same category as at baseline. Mediterranean diet adherence was determined using the Mediterranean Diet Adherence Screener (MEDAS [33]). High adherence to the Mediterranean diet was defined as having a score ≥8. Depressive symptoms were assessed with the Patient Health Questionnaire 9 (PHQ-9). A score of ≥10 was used as cut-off [34]. Health conditions (hypertension, dyslipidemia, diabetes, coronary heart disease, and chronic kidney disease) were based on self-reported history of diagnosis and patient health record data. Participants’ blood pressure was measured during the baseline consultation. Measurements of blood lipids, glycemia, and blood pressure at follow-up were also collected if they were available through the participants’ health records. Measurements were considered as baseline measurements if they were collected maximum 6 months before the consultation date and up to two weeks afterwards. Follow-up measurements were considered if they were collected between two weeks after the baseline consultation and up to one year later. A practice nurse of the general practice assembled data on the above listed health conditions and measurements of the participants and transferred these to the research team.

#### Knowledge on dementia risk reduction

Knowledge about dementia risk reduction was examined via 13 statements (see Supplementary Material 2) which have been used previously [35–38]. Participants could respond on 5-point Likert scale, ranging from “completely disagree” to “completely agree”. Lack of awareness of the potential for dementia risk reduction was defined as agreeing to the statement “There is nothing one can do to reduce their risk of getting dementia”. All other statements surveyed knowledge of specific modifiable dementia risk and protective factors. Knowledge of dementia risk reduction was operationalized by assigning scores to each of the 13 statements and taking the sum. With “complete agreement” to a correct statement, four points were assigned to the participant’s score. With “agreement”, three points were assigned, and so on. Complete disagreement to a correct statement led to zero points being assigned. For the general (incorrect) statement on dementia risk reduction, the scoring was the inverse to that described above.

#### Other measures

Custom items on lifestyle changes, facilitators and barriers towards it, and social-cognitive determinants of behavior (i.e., attitude, self-efficacy, and intention for a healthy lifestyle) were included in the online surveys. The follow-up survey also probed for experiences with, and perspectives on, the PCP consultation, the discussed personal dementia risk reduction profile, and the MyBraincoach app, in order to assess acceptability and demand for the intervention. In addition, objective app usage data was available as the number of activated themes and the total opened daily notifications in MyBraincoach app. Participants in the app-supported arm were categorized into low intensity users and high intensity users based on median split by total opened daily notifications.

### Interviews

Seven interviews with participants (all from the app-supported arm) were conducted to get an in-depth view of their experiences and examine the acceptability of the different intervention components. In addition, four interviews with PCPs who carried out baseline consultations, were conducted to assess demand, integration, and (potential) implementation of the intervention from their perspective. Overall, the objective was to conduct a process evaluation of what parts of the intervention worked well, what did not, and why, as well as to explore potential improvements for the future. Interviews were done face-to-face or via video call (on Zoom virtual meeting platform), depending on the interviewee’s preference. They were semi-structured and took approximately 30–50 minutes. All interviews were audio recorded and later transcribed. The resulting transcript was coded and analyzed based on the grounded theory, using Atlas.ti, a qualitative data analysis software. Within the grounded theory, data is explored without predefined theory or concepts, allowing for a more free exploration of the constructs of interest [39]. Interview guides are included in Supplementary Material 3.

### Statistical analysis

Population descriptive characteristics were compared between the consultation-only and app-supported arm using χ^2^ tests or Fisher’s exact tests (in case of cells with *n* < 5) for categorical outcomes, and unpaired *t*-tests for continuous outcomes. Further, sociodemographic characteristics, knowledge of dementia risk reduction, social-cognitive determinants of health behavior, and prevalence of modifiable risk and protective factors were compared between low and high app users, using χ^2^ tests and unpaired *t*-tests.

Considering the cluster (practice-level) randomization, linear mixed models were used to analyze changes over time between study groups (time×study group estimate). Specifically, three-level linear mixed models with a random intercept for time nested within individuals nested within general practices were used. Fixed effects estimates and their associated *p*-values are shown. Additionally, changes over time in binary outcomes (e.g., adherence to a Mediterranean diet (yes/no), or obesity (yes/no)) were explored using logistic mixed model analysis. Mean and SD, or median and interquartile range (IQR) are given, depending on the data distribution. All tests were carried out two-sided with an alpha level of 0.05. All analyses were conducted with Stata 17.0 (StataCorp, College Station, Texas, United States).

## RESULTS

### Population

**Fig. 3:**
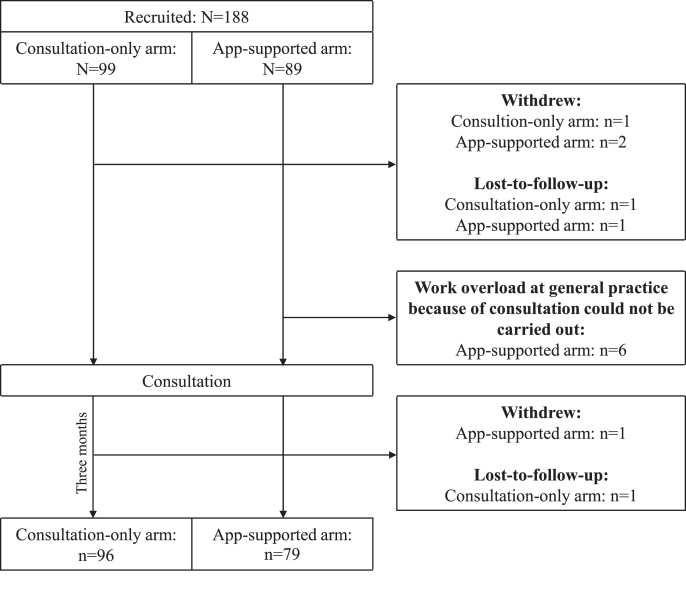
Trial profile.

In total, 188 participants were recruited (consultation-only *n* = 99, app-supported *n* = 89). Three app-supported arm participants withdrew, and one was lost to follow-up. Reasons for withdrawal included dissatisfaction with the consultation, lack of time, and unspecified health issues. An additional six participants could not complete the study due to work overload at their general practice. One participant in the consultation-only arm withdrew (due to lack of time), and two were lost to follow-up. Finally, 175 completed the study and were included in the analytical sample (Fig. 3).

Detailed population characteristics are listed in Table 1. Participants who withdrew or were lost to follow-up but still completed the baseline questionnaire (*n* = 8) were compared with individuals in the analytical sample. Individuals who did not complete the study were more often low educated (*p* = 0.001), more often had depressive symptoms (*p* = 0.032), but fewer had elevated cholesterol levels (*p* = 0.044). No other differences were observed.

**Table 1:** Baseline population characteristics

Consultation-only	App-supported	p
Total N	96	79
Age, mean (SD)	52.8 (5.3)	53.9 (5.8)	0.163
Female sex, *n* (%)	53 (55.2)	45 (57.0)	0.816
Educational level^1^, *n* (%)	0.368
Low	4 (4.2)	2 (2.5)
Intermediate	47 (49.0)	47 (59.5)
High	45 (46.9)	30 (38.0)
Marital status^1^, *n* (%)			0.320
Married or registered partnership	60 (62.5)	59 (74.7)
Living together	10 (10.4)	8 (10.1)
Single	11 (11.5)	7 (8.9)
Divorced	13 (13.5)	4 (5.1)
Widowed	2 (2.1)	1 (1.3)
Neighborhood socioeconomic status (SES)			0.583
Low SES neighborhood	35 (36.5)	23 (30.7)
Middle SES neighborhood	32 (33.3)	24 (32.0)
High SES neighborhood	29 (30.2)	28 (37.3)
Lifestyle factors, *n* (%)
Physically inactive	38 (39.6)	28 (35.4)	0.574
Adherent to Mediterranean diet	10 (10.4)	6 (7.6)	0.519
Smoking	12 (12.5)	7 (8.9)	0.441
Low-to-moderate alcohol consumption	81 (84.4)	68 (86.1)	0.753
High cognitive activity	29 (30.2)	26 (32.9)	0.701
Health conditions, *n* (%)
Depressive symptoms^1^	5 (5.2)	4 (5.1)	1.000
Hypertension	35 (36.5)	46 (58.2)	**0.004**
Diabetes	6 (6.3)	7 (8.9)	0.512
Dyslipidemia	54 (56.3)	40 (50.6)	0.458
Obesity	26 (27.1)	24 (30.4)	0.631
Coronary heart disease	19 (19.8)	14 (17.7)	0.728
Chronic kidney disease^1^	3 (3.1)	4 (5.1)	0.703
LIBRA score, mean (SD)	0.863 (2.6)	1.061 (2.6)	0.612
Knowledge on dementia risk reduction (sum score), mean (SD)	32.1 (7.2)	33.3 (5.7)	0.248

Tested using χ^2^ test for binary outcomes and unpaired *t*-test for continuous outcomes. ^1^tested using Fisher’s exact test. SD, standard deviation; LIBRA, LIfestyle for BRAin health.

### Participants’ perspectives on the different intervention components

Generally, views on the modifiable dementia risk (LIBRA) profile, discussed during the face-to-face consultation with the PCP, were positive. Specifically, 116 (66.3%) participants found the profile itself insightful and 126 (72.0%) found it interesting to discuss it with their PCP. Ninety-five participants (54.3%) reported the profile to be motivating, but 31 (17.7%) also indicated it was confronting.

**Fig. 4:**
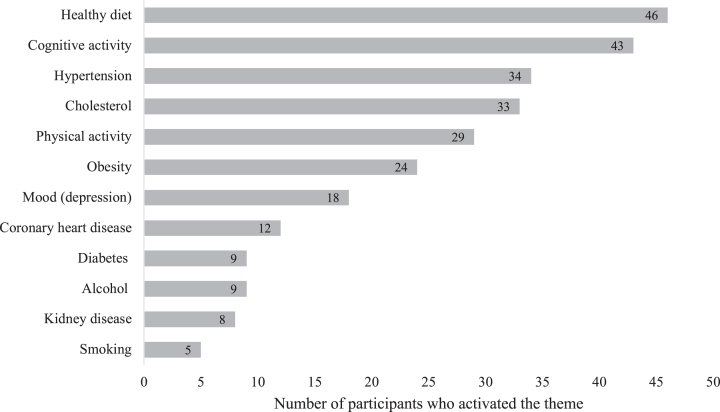
MyBraincoach topics activated by participants in the app-supported arm (activation of multiple topics over time possible).

For the MyBraincoach app, 71 (89.9%) of participants in the app-supported arm installed the app as asked. Of these, 65 (91.6%) reported they also used it. According to the app usage data, 9 (11.4%) participants in the app-supported arm activated one risk factor topic. Another 12 (15.2%), 13 (16.5%) and 10 (12.7%) activated two, three and four topics, respectively. An additional 17 participants (21.5%) activated between five and seven topics and, 6 (7.6%) participants activated eight to twelve topics. Twelve participants (15.2%) did not activate any topic and, as a consequence, also did not open any daily notifications. For the other participants, the total number of opened daily notifications was highly skewed with a median of 42 (IQR = 45, range = 2–168). The most popular topic was healthy diet (activated by 46 participants (58%)), followed by cognitive activity and hypertension. The number of participants that activated a topic is shown in Fig. 4.

The app was generally positively perceived as being clear, informative, nice looking, enjoyable, and easy to use. Specifically, 45 (69.2%) of participants who used the app found its tips and advice useful, and 43 (66.2%) indicated the information within was personally applicable. Moreover, 38 (58.5%) of the app users reported the app helped them to work on their lifestyle. The app got scored a 7.4 out of 10 by its users (SD = 1.6) with no differences between demographic groups.

### Change in LIBRA over time

**Fig. 5:**
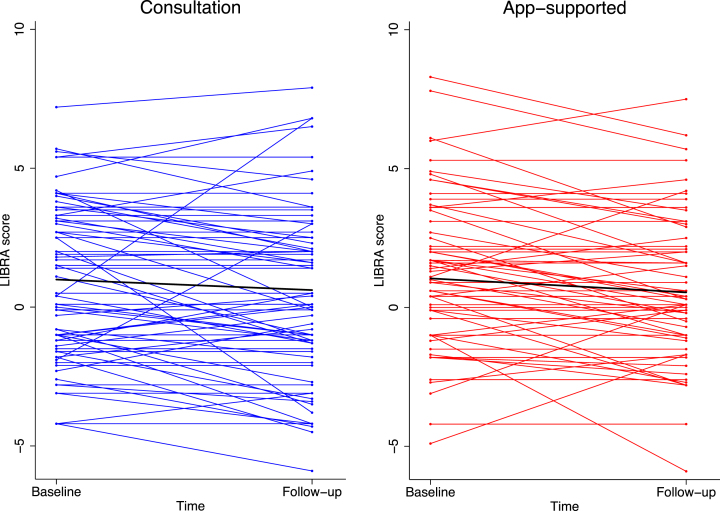
Change in the Lifestyle for Brain Health (LIBRA) score over the three-month study period in the two intervention arms at the individual and mean level (thick black line).

LIBRA significantly improved over time in the total sample (–0.44 [95% confidence interval (CI) –0.66 to –0.21], Fig. 5). There was no difference between the two intervention arms over time (time×intervention group estimate: –0.12 [95% CI –0.57 to 0.32]).

Sociodemographic characteristics predictive of change over time in LIBRA in the total sample were explored. A higher level of education was associated with a better (i.e., lower) baseline LIBRA score (intermediate versus low educated: –2.13, *p* = 0.032; high versus low educated: –3.10, *p* = 0.002) but also with less improvement over time (intermediate *versus* low educated:+1.77, *p* = 0.004; high versus low educated:+1.94, *p* = 0.002). No other characteristics were associated with LIBRA change over time but participants living in a higher SES neighborhood tended to have a better baseline LIBRA score (high versus low SES neighborhood: –1.08, *p* = 0.037). As more individuals in the app-supported arm had hypertension at baseline, a sensitivity analysis adjusting for hypertension was performed. Results were similar to the primary analyses.

The association between app usage and evolution in LIBRA was examined in the app-supported group. Each additional “nut cracked” was associated with an extra improvement (i.e., decline) in LIBRA of –0.01 over time (*p* = 0.024). For each additional activated topic, an additional improvement in LIBRA of –0.11 was estimated, although not significant (*p* = 0.063). Low and high intensity app users did not differ from each other based on age, sex, level of education, and baseline measure of intention, attitude, or self-efficacy for a healthy lifestyle. However, individuals who used the app much had a higher cognitive activity score (127 versus 117, *p* = 0.033) at baseline, but were more often physically inactive (21.9% versus 44.7%, *p* = 0.038).

### Change in individual modifiable risk and protective factors

The physical activity level of both intervention arms improved over time (+0.11 [95% CI 0.01–0.22]) without a difference between the two. In terms of Mediterranean diet adherence, the MEDAS score improved in the entire sample (+0.69 [95% CI 0.45–0.92]). Moreover, more individuals were adherent to the Mediterranean diet at follow-up compared to baseline (odds ratio = 3.78, *p* = 0.008). At follow-up, the number of tobacco products smoked per week was estimated at 2.8 less compared to baseline, although this difference was not significant ([95% CI –5.67 to 0.13]). BMI decreased over time (–0.21 kg/m^2^ [95% CI –0.35 to –0.06]) but there was no study group difference. Blood lipid and glycemia measures were not available for many of the participants, and in them, they did not change over time. There were no changes in cognitive activity, blood pressure, alcohol consumption, or depressive symptoms over time in the total sample or differences therein between study groups.

### Knowledge on dementia risk reduction and social-cognitive determinants of health behavior

**Fig. 6:**
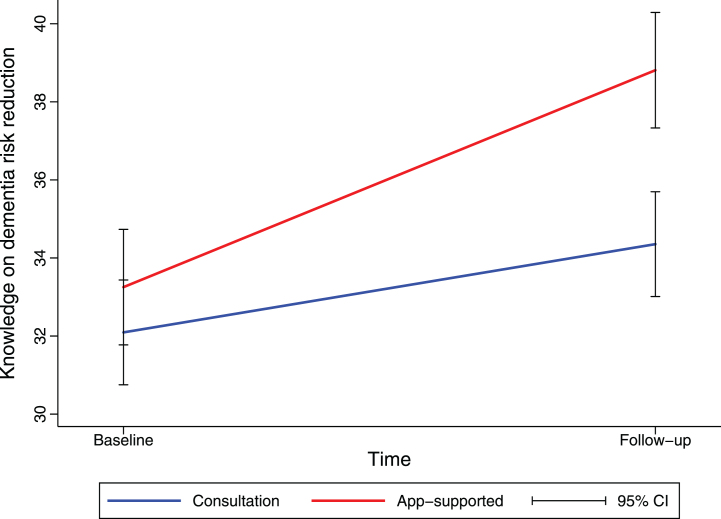
Knowledge on dementia risk reduction increased more in the app-supported group. Mean change in knowledge on dementia risk reduction from baseline until follow-up (at three months), as estimated by linear mixed model analysis, is shown. Values for knowledge are sum scores based on a 13-item questionnaire. CI, confidence interval.

Knowledge on dementia risk reduction significantly increased over time (+2.26 95% CI 1.01–3.51]), and more so in the app-supported group than in the consultation-only group (+3.30 [95% CI 1.43–5.16], Fig. 6). Further, the total number of topics a participant activated within the MyBraincoach app (+1.78 [95% CI 1.13–2.43]) and the total number of daily notifications they opened (+0.12 [95% CI 0.08–0.17]) was positively associated with the knowledge increase over the course of the study. There were no other changes over time in the total sample, nor dependent on study group in terms of intention for a healthy lifestyle, attitude towards a healthy lifestyle, or self-efficacy herein.

### Participant interviews

#### General view on participation and discussion of the dementia risk factor profile

Most interviewed participants indicated they were happy to have participated and found participation easy. On the content of the intervention, opinions differed. Some participants found the information they received during the consultation and within the app interesting, whereas a few others found the information too basic and indicated they did not learn a lot of new things. A couple of participants also mentioned they would have liked more information on dementia itself, its early signs, and the current “condition” of their own brains. Regarding the consultation with the PCP, most interviewees indicated they were satisfied with its content. However, even though the PCPs were instructed to set personal lifestyle goals with the participants during the consultation, some of the interviewed participants said this did not happen and they instead got more general advice. This appeared less valuable to them. Other participants emphasized that it is important to them to take their personal situation, values, and goals into account during such consultations. They also mentioned that discussing dementia risk reduction with their PCP resulted in an increased awareness and gave them an extra stimulus to make lifestyle improvements.

(male, high education): “*And that is indeed another contribution. You hear again* ‘*exercise is good*’, ‘*moving three times a week is good*’, ‘*eating healthy is good*’, ‘*what is healthy eating*’, ‘*what do you eat then: a lot of vegetables, a lot of fruit, you know, not too much fruit because there are a lot of sugars in there*’*, that kind of stuff. All that information, you indeed become aware of again.* ‘*Keep it up.*’ *And I was already occupied with that, but it did give me an extra push to keep up.*”

Most participants indicated they enjoyed the personal contact, and many appreciated a scheduled follow-up consultation. Almost all interviewed participants who did not get offered a follow-up consultation mentioned that they would have liked this to monitor their status and evaluate any efforts made, ideally six to twelve months after the initial consultation.

 (male, high education): “*What I missed is another check-up with the GP. To discuss the progress, like* ‘*how is it going?*’, ‘*stick to your goals*’*. Because now, it just was that app, and alright, there are goals in there, which you try to focus on, but I think for some people, for me as well, an incentive to keep this commitment is needed, and you need a follow-up consultation to make sure things are happening.*”

Other suggested improvements included regular health check-ups from a certain age and the provision of a small report of what was discussed during their consultation to take home.

#### The MyBraincoach app

On the smartphone app, most participants felt like it helped them to stay focused on their lifestyle goals, even if the info within the app was already known. They said the app offered nice little nudges or triggers after the initial consultation with the PCP.

(male, intermediate education): “*Yes exactly, I always used to think it just happens to you, but actually you can do something yourself with your lifestyle, so that if you do get it, that it happens as late as possible.*”

Some participants suggested that further personalization of the app would be nice. Some wanted more tips, recipes, or indicated that a video or more visual content would be appreciated. Two interviewees mentioned they would like to monitor their lifestyle within the app, along with advice that matches this personal data, or the potential benefit that you may attain if you make certain changes. Lastly, some participants wanted a more general theme on dementia to be included in the app.

#### Lifestyle changes

Certain participants reported they made lifestyle changes because of, or supported by, the intervention. One interviewee bought a blood pressure monitor, another improved their diet (e.g., more olive oil, less meat, more fish, more nuts), and yet another implemented more moments for relaxation.

 (male, intermediate education): “*We actually, when we got the app, I looked at it together with my wife and it indicated that body weight was a theme for us. So, we looked together what this actually means. Because of this, we said* ‘*let*’*s pay some more attention to our diet*’*, to live a bit healthier.*”

 (female, intermediate education): “*Now I always use olive oil to fry things and I also started using [a brand of margarine]. It*’*s more expensive but has omega 3. Also, moving a bit more. My husband doesn*’*t have high cholesterol anymore. I get my results in a couple weeks and I*’*m curious if mine also improved.*”

Some interviewees indicated they felt as if the app gave them an extra stimulus to change a certain behavior as they were now aware it was also beneficial for their brain health. However, some interviewees also reported they did not make any lifestyle changes.

#### Barriers and facilitators for behavior change

Participants mentioned several facilitators and barriers for lifestyle changes. Facilitators included professionals support, support from the people around them, accurate risk perception, intrinsic motivation, more free time, and seeing progress. Aging was also mentioned as a facilitator, as some participants indicated they became more aware of their health, dementia, and asked themselves what they still wanted to do with their lives, which according to them could trigger positive change. Several interviewees also mentioned that follow-up visits, during which your personal progress is evaluated, are helpful towards change. Lastly, discipline or “just doing it” was mentioned often. Reported barriers for behavior change were health problems, financial limitations, lack of knowledge and awareness, stress, lack of time, and lack of support from the personal environment.

 (male, high education): “*Yes, we are currently busy starting up a factory. So, with that comes a bit of stress of course. I do notice that in my diet. So, if I have a lot of stress, I*’*ll choose less good food options more often, and I*’*ll definitely eat more.*”

### Healthcare professional interviews

#### General view on discussing dementia risk reduction during a consultation

None of the four interviewed PCPs had discussed dementia risk reduction or brain health with patients before this study. They reported the consultation took about 20–30 minutes, and this worked well for them, although two PCPs also pointed to a general lack of time. The PCPs’ experiences were overall positive. Especially the fact that the modifiable risk factors for dementia are already routinely discussed as part of cardiovascular risk management resulted in them concluding that it would be of added value to bring up brain health, and easy to implement this in primary care. Two PCPs specifically mentioned that they already discussed all the themes relevant for brain health promotion, just without referring specifically to the fact that this may also lower the risk for dementia.

“*No, I don*’*t think it would take up a lot of additional time if it [the topic of brain health] gets added to our standard discussion points. I already ask patients routinely about their social contacts, loneliness, hobbies. I actually already discuss these topics. So, I think it would be a small effort to take on the topic of brain health.*”

Three PCPs mentioned they noticed improvements in their participating patients in terms of behavior, motivation, or awareness. One PCP reported one of their patients with diabetes finally agreed to start with insulin, and because of this now has good glycemic values. The PCP thought that participation in this study and the mention that good glycemic control is beneficial for the brain, may have triggered this change. Two PCPs emphasized the increased awareness of their patients.

“*This also opened people*’*s eyes a little bit. You know, people would respond like* ‘*Oh yes, I don*’*t engage in physical activity that much*’ *or* ‘*I should read a book a bit more often.*’ *Yes, it inspired them.*”

#### Needs and wishes for effectively discussing dementia risk reduction with patients

Three PCPs reported they appreciated the brain health profiles that were made for them to use during the consultation as it facilitated the conversation. In general, they find conversation starters, especially ones that are visual and simple, very helpful. One PCP said her patients sometimes did not recognize themselves in the brain health profile, which then made the consultation more difficult. Two PCPs emphasized the general importance of being face-to-face with a patient to properly connect with them to facilitate behavior change. In addition, follow-up visits were important according to two PCPs to continue monitoring and evaluate progress.

Two PCPs mentioned they often give their patients something to take home. Two PCPs liked online materials such as an app or website, whereas one other PCP preferred hard-copy materials. One PCP specifically reported that a visual chart to be used during the consultation would be very useful, aiming at improving risk perception, what can be done to reduce risk, and even better if it can show the risk reduction in a more tangible way (e.g., disease free life years). When asking the interviewees what they thought about a brain health promotion smartphone app, all PCPs were very positive about referring their patients to an app like this and thought their patients would be interested to use it. One PCP added she thought apps were especially used by more highly educated patients and less so by low-educated patients, who according to her, preferred simple, visual printed materials.

 “*But that would be really nice for us to know that this app exists, we can download it and then have a look and see if it may be fitting for some people and then we can offer this [to patients].*” 

All PCPs indicated they would like to receive education on the topic of dementia risk reduction. For example, covering how important genetics are within the total risk, or how important each modifiable risk factor is. They prefer this education to be face-to-face in group as this allows them to discuss what is learned with colleagues.

#### Discussing dementia risk reduction in general practices in low socioeconomic settings

One interviewed PCP worked at a general practice in a low socioeconomic setting. Her view differed from the other interviewed PCPs. According to her, implementation of brain health promotion or dementia risk reduction in the practice where she worked would be overly ambitious. Specifically, she stated that her colleagues and she were already happy if their patients showed up for their appointments or took their medication as prescribed. According to her, looking further ahead with something like dementia risk reduction aims too high, as a lot of their patients simply have other issues or struggles that make it difficult to care about things (far) in the future.

 “*Yes, there are people with a debt management plan, people without a job, people going through a divorce, people from different cultural background so that makes things very different then. They don*’*t think beyond the now.*”

## DISCUSSION

The current cluster-randomized study showed that discussing risk reduction for dementia during regular primary care consultations is feasible and can be effective in improving the risk profile of patients in this middle-aged population. The intervention was effective at improving knowledge of dementia risk reduction. Acceptability of the different intervention components (PCP consultation, mHealth support) was good. Awareness about dementia risk reduction was still largely lacking at the start of the intervention, and there appears to be a demand for information on this topic. After the PCP consultation, participants significantly improved on a validated modifiable dementia risk score over the three-month-long study period. Additionally, knowledge of dementia risk reduction increased over the study period, and more so in participants who got offered the brain health promotion smartphone app. Additional workload for PCPs appeared limited, and according to them, discussing brain health, or dementia risk reduction, could constitute an effective additional stimulus for behavior change.

We observed significant improvements on multiple outcomes over the study period in the total sample. LIBRA significantly decreased in both study arms, with an average 0.44 points. Previous observational research has demonstrated that a one-point decrease in LIBRA is associated with 6–16% lower risk for incident dementia [16, 22, 23, 26]. However, only knowledge about dementia risk reduction improved significantly more in the group who also received the MyBraincoach app. Indeed, the app was designed to increase knowledge and awareness of dementia risk factors, but with the ambition that this affects other social-cognitive determinants of health behavior further downstream, and finally health behavior itself [40]. The study period of three months might have been too short to capture these, as it is possible that it takes more time before these downstream effects on behavior become visible. Moreover, the consultation received by both study arms may have been sufficient for behavior change, so that additional provision of the smartphone app did not have an added value in terms of tackling lifestyle factors. In addition, while it is encouraging to see that a relatively simple, low-intensity intervention led to positive results, we cannot rule out that improvements in health behavior occurred naturally in a group of individuals interested and motivated to participate in a study on brain health promotion (Hawthorne effect). Two previous large RCTs that aimed at dementia risk reduction and ran via primary care, found no positive effects of the tested lifestyle intervention on their primary outcomes (dementia incidence, disability, and cognitive performance) [41, 42]. Being a feasibility study, we did not assess these health-related or cognitive outcomes but instead examined preliminary efficacy via changes in modifiable risk and protective factors. However, there are several potential explanations for the negative results of these trials, including the age of the participants and the high-quality care within the control groups. A recent systematic review and meta-analysis showed that it is very difficult to achieve behavior change among low socioeconomic groups using mHealth or educational interventions [43]. Interestingly, lower educated individuals showed more improvement on the LIBRA score over time than higher educated individuals in our study. There is no clear explanation for this, besides the fact that the ‘MyBraincoach’ app is typically valued more by lower educated individuals, although app ratings did not differ based on educational level in this sample [18]. Lower educated individuals, also, on average, had worse LIBRA scores at baseline and thus more room for improvement than those higher educated.

Our results also suggest that discussing dementia risk reduction in primary care, next to cardiovascular risk management, may be feasible. The drop-out rate was low (6.9% 25;). The PCP consultation and the app were generally well received and appreciated. We previously showed that there is a considerable demand for information on dementia risk reduction in the middle-aged population [35, 36]. Many middle-aged individuals are at least distantly acquainted with dementia and are becoming more conscious of aging-related conditions. Raising awareness and increasing knowledge is indeed an important first step towards conscious behavior change [44]. It is therefore important to discuss the risk profile and cues to action in PCP consultations. Importantly, PCPs involved in the study almost unanimously reported that it would be easy to implement the discussion of brain health in primary care along with advising on, and monitoring, of the modifiable risk and protective factors. They also agreed that it would be of added value to mention brain health in their role as health advisor. Indeed, findings from a qualitative study in general practitioners (GPs) from the United Kingdom also highlighted that GPs consider dementia risk reduction to fall within their scope [45]. Guidelines on how this can be done have been published [15]. Nonetheless, there are certain potential barriers that should be considered. Limited time and resources being the major ones, that may force PCPs to neglect efforts towards prevention, despite their intentions, as it is usually less of priority or urgent [45, 46]. Dutch general practitioners for example do not get reimbursed for dedicating time towards discussing prevention with their patients. Additional education (e.g., educational meetings) for PCPs on the topic of dementia risk reduction is also recommended and is called for by PCPs themselves [45–48].

Within the smartphone app, especially the format of the daily notification appeared to be appreciated and beneficial towards supporting focus and motivation for behavior change. However, certain potential improvements were noted, such as offering more information to those who are especially interested, perhaps via links to other more in-depth sources, while also not overburdening those who are less interested. More personalization and/or self-monitoring of behavior within the app were also suggested in the interviews, which is in line with current knowledge on mHealth for behavior change [49]. Although smartphones are used by a large majority of the global population and have been suggested to constitute a potential solution to reach individuals with a low socioeconomic position with health messages, health app usage has been associated with higher levels of education and less socioeconomic deprivation [50–52]. Therefore, it is not entirely clear whether mHealth technologies may decrease or increase health inequalities [50]. In any case, it is important to tailor mHealth technologies to individuals who have low health literacy and/or have a lower socioeconomic position, or to provide other tools suited to their needs and preferences, to ensure we are not increasing health inequalities even further [50].

Important strengths of the current study include the cluster-randomized study design which prevents potential contamination bias, and the variety in size and socioeconomic setting of the general practices which benefits external validity. Various measures important to feasibility were assessed, and the conducted interviews provided valuable insights for process evaluation. The current study also had certain limitations. Being a proof-of-concept study, it was relatively small, and most outcome measures were questionnaire-based. Most questionnaires were validated but social desirability still could have played a role [53]. LIBRA scores improved in both groups, and since we lacked a true control or care-as-usual group, we cannot rule out that this was due to study participation rather than the intervention components. Follow-up was done at three months which is rather short and means that little can be said about the sustainability of the lifestyle changes that were made. Further, study participants are often especially motivated and interested individuals, potentially inflating the current positive findings. PCPs of the participating general practices could also be especially motivated and interested in prevention, which limits external validity. This may also have affected the representativeness of their views on the intervention. Participants were financially compensated for their time investment and travel costs, whereas general practices were compensated for their personnel costs and use of consultation rooms. This could have led to biased enrollment. However, financial compensation in this study was quite low and participants’ socioeconomic position (as assessed via level of education and neighborhood level SES) was relatively high. Future work investigating the potential of this intervention on a larger scale is necessary, including a control group (i.e., care as usual), longer follow-up duration, and more objective measurements of relevant outcomes including sustained risk profile changes and cognitive status.

Taken together, the current proof-of-concept feasibility study suggests that discussing brain health in general practice, perhaps using a smartphone app to further promote brain health, may be a feasible and effective, scalable approach to reduce the risk of dementia. However, further confirmatory work is needed to substantiate this.

## AUTHOR CONTRIBUTIONS

Stephanie Van Asbroeck (Conceptualization; Data curation; Formal analysis; Investigation; Methodology; Project administration; Visualization; Writing – original draft); Sebastian Köhler (Conceptualization; Methodology; Supervision; Writing – review & editing); Sophie Wimmers (Investigation; Writing – review & editing); Jean Muris (Resources; Writing – review & editing); Martin van Boxtel (Conceptualization; Methodology; Supervision; Writing – review & editing); Kay Deckers (Conceptualization; Funding acquisition; Methodology; Project administration; Supervision; Writing – review & editing).

## Supplementary Material

Supplementary Material 1

Supplementary Material 2

Supplementary Material 3

Supplementary Material 4

## Data Availability

The data collected and used in this study is available upon reasonable request from the corresponding author. The metadata and codebook can be freely consulted [54].
