# Supplementary material for: Multidomain Dementia Risk Reduction in Primary Care is Feasible: A Proof-of-concept study
Source: J Alzheimers Dis. 2024 Jun 11;99(4):1455–71. doi: 10.3233/JAD-240229 (PMC11191460; doi:10.3233/JAD-240229)
Supplement: Supplementary Material 1 [file jad-99-jad240229-s001.docx]

# modifiable dementia risk profile

| **Modifiable** | |
| --- | --- |
| 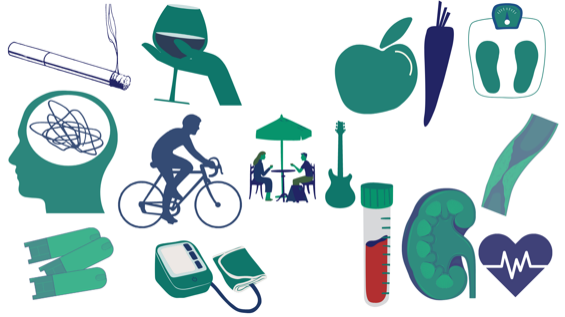  Cognitive activity | Great! |
| 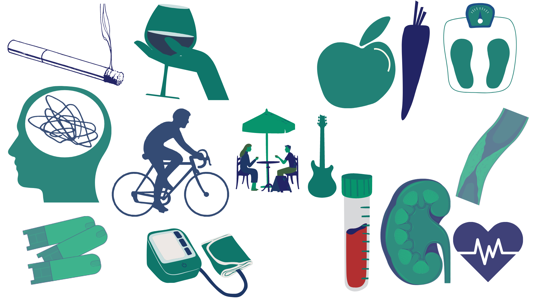  Physical activity | Great! |
| 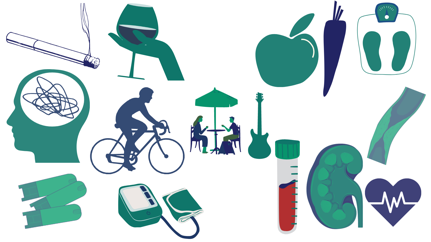  Diet | **Room for improvement,** potential changes include eating more legumes and reducing the consumption of soft drinks and sweets. |
| 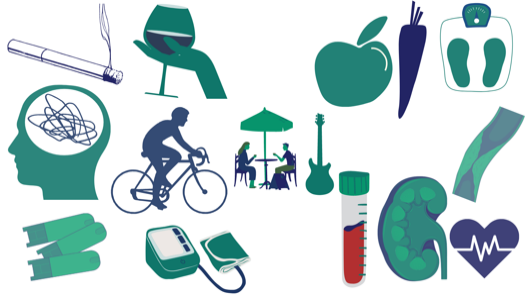  Alcohol consumption | Great! |
| 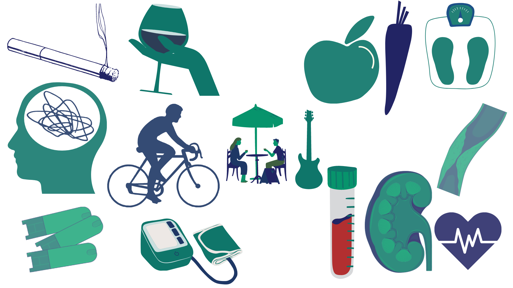  Tobacco | Great! |
| **Modifiable (to a certain extent) in the long term** | |
| 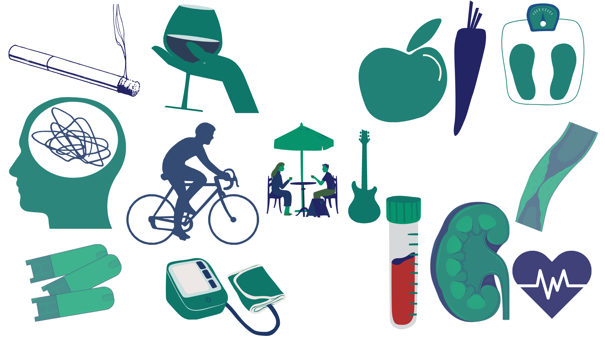  Obesity | **Room for improvement** |
| 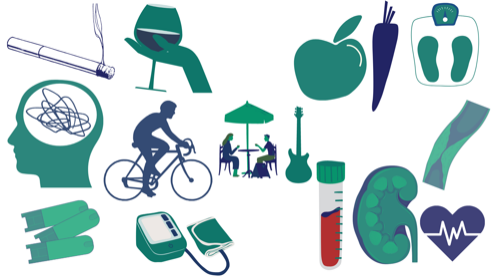  Mental wellbeing (depression) | **Room for improvement** |
| 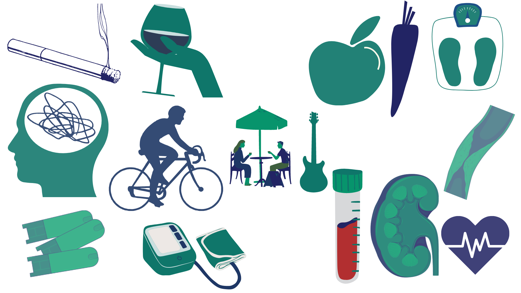  Elevated blood pressure | **Room for improvement** |
| 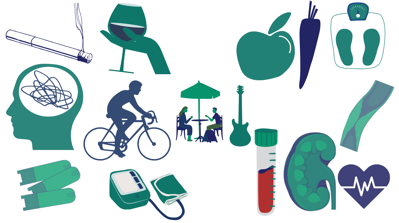  Elevated cholesterol | Great! |
| **To keep an eye on** | |
| 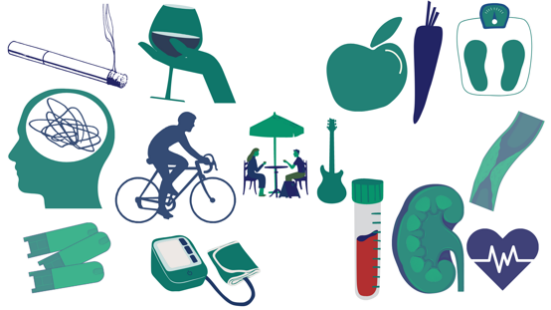  Diabetes | Great! |
| 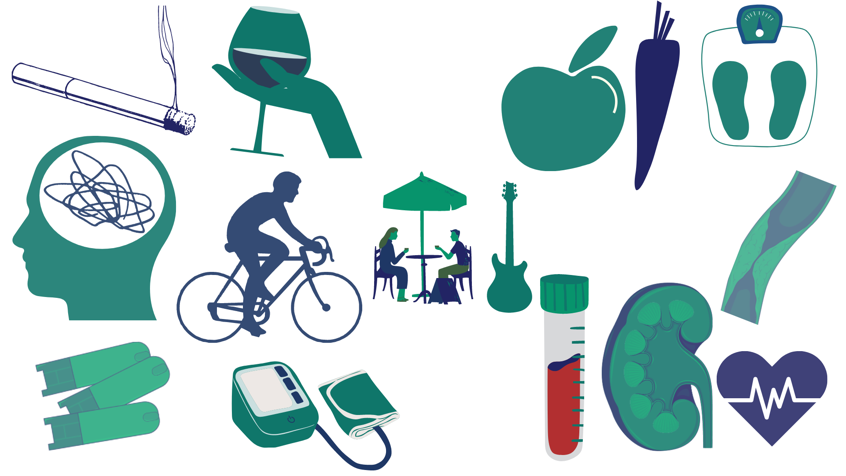  Coronary heart disease | Great! |
| 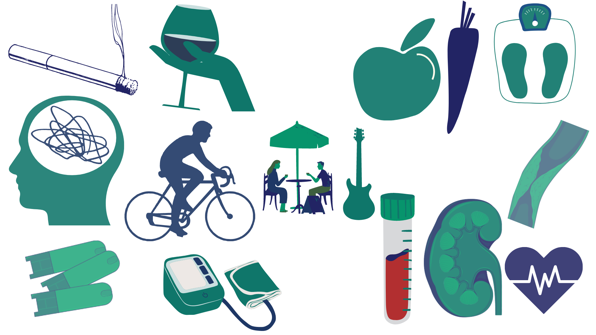  Chronic kidney disease | Great! |
