# Supplementary material for: Multidomain Dementia Risk Reduction in Primary Care is Feasible: A Proof-of-concept study
Source: J Alzheimers Dis. 2024 Jun 11;99(4):1455–71. doi: 10.3233/JAD-240229 (PMC11191460; doi:10.3233/JAD-240229)
Supplement: Supplementary Material 2 [file jad-99-jad240229-s002.docx]

1. **Screening and baseline survey**
2. What is your date of birth?
3. Do you have a smartphone? This is a mobile phone on which you can use apps.

- Yes
- No

1. Do you have (regular) access to the internet with your smartphone?

-
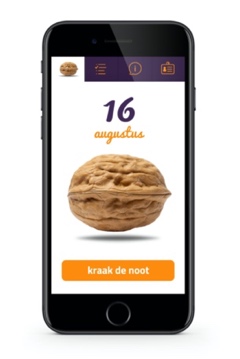
Yes
- No

1. Have you ever used the MijnBreincoach app (see screenshot)?

- Yes
- No

1. Which general practitioner's practice do you go to?

[List of participating practices]

1. What is your sex?

- Male
- Female

1. What is the highest degree or level of school you have completed?

[Six categories according to the Dutch education system]

1. What is your marital status?

- Married/registered partnership
- Living together
- Unmarried, never been married
- Divorced
- Widowed

1. What is your postal code?
2. What is the estimated gross income of your household per month?
3. Are you currently being supported or followed-up by a dietician or lifestyle coach?
   - Yes
   - No
4. What is the operating system of your smartphone?

- iOS (iPhone)
- Android
- Other
- I’m not sure

1. How often do you consume alcoholic beverages?

- Never
- Maximum once per month
- 2-4 times per month
- 2-3 times per week
- 4 or more times per week

1.
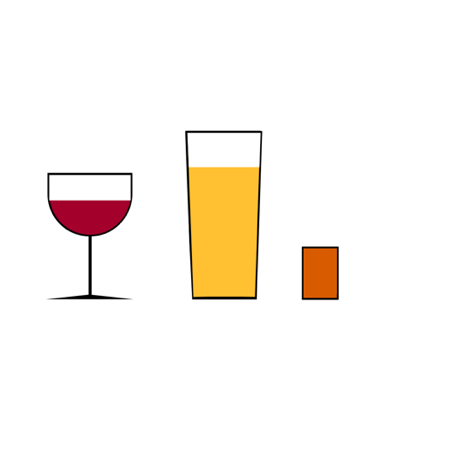
[If answer to question 6 is not "never"] On the days when you drank alcohol in the past year, how many standard glasses did you typically consume? A standard glass is 25 cl of beer (4-5%), a small glass of wine, or a shot of strong liquor.

- 1-2 standard glasses
- 3-4 standard glasses
- 5-6 standard glasses
- 7-9 standard glasses
- 10 or more standard glasses

1. Has your doctor ever told you that your cholesterol is too high?

- Yes
- No

1. Has your doctor ever told you that you have a heart or vascular condition (e.g., heart attack, angina, heart failure, stroke, and TIAs)?

- Yes
- No

1. Has your doctor ever told you that you have high blood pressure?

- Yes
- No

1. Has your doctor ever told you that you have diabetes?

- Yes
- No

1. Has your doctor ever told you that you have a chronic kidney disease?

- Yes
- No

*Mediterranean diet adherence screener (MEDAS) items [1]*

1. Do you use olive oil as main culinary fat?

- Yes
- No

1. How much olive oil do you consume in a given day (including all oil used for frying, salads, out-of-house meals, etc.)?

- 0-1 tablespoon
- 2-3 tablespoons
- 4 or more tablespoons

1. How many serving spoons of vegetables (cooked and raw combined) do you eat per day?

- 0-3 serving spoons
- 4-7 serving spoons
- 7 or more serving spoons

1. How many fruit units (including natural fruit juices) do you consume per day? One unit of fruit is for example one average sized apple, one average-sized banana, two little mandarins, seven strawberries or one glass 100% natural fruit juice.

- 0 units
- 1 unit
- 2 units
- 3 or more units

1. How many servings of red meat or meat products (e.g., beef, pork, minced meat, sausage, game meats; but no poultry like chicken or turkey) do you consume per day? One serving is 100-150 g or the size of a deck of cards.

- Less than 1 serving
- More than 1 serving

1. How many servings of butter, margarine, or cream do you consume per day? One serving is about 1 tablespoon.

- Less than 1 serving
- More than 1 serving

1. How many sweet or carbonated beverages do you drink per day?

- Les than 1 glass
- 1 glass or more

1. How much wine do you drink per week?

- 0-6 glasses
- 7 or more glasses

1. How many servings of legumes (e.g., brown beans, white beans, peas, lentils) do you consume per week? 1 serving is 150 g or 3 tablespoons.

- 0-2 servings
- 3 or more servings

1. How many servings of fish or shellfish do you consume per week? 1 serving is 100-150 g or a medium-sized tin of salmon, tuna, or sardines.

- 0-2 servings
- 3 or more servings

1. How many times per week do you consume commercial sweets or pastries, such as candy, cakes, cookies, biscuits, or a sweet desert?

- 0-2 times
- 3 or more times

1. How many servings of nuts (including peanuts) do you consume per week? One serving is one handful.

- 0-2 servings
- 3 or more servings

1. Do you preferentially consume chicken, turkey, or rabbit instead of veal, pork, hamburger, or sausage?

- Yes
- No
- Not applicable

1. How many times per week do you consume dishes with sofrito (sauce made with tomato and onion, leek, or garlic, and olive oil?)

- 0-1 time
- 2 times or more

*Patient health questionnaire 9 (PHQ-9) items [2]*

Over the last 2 weeks, how often have you been bothered by any of the following problems?

[Answer options:

- Not at all
- Several days
- More than half of the days
- Nearly every day]

1. Little interest or pleasure in doing things
2. Feeling down, depressed, or hopeless
3. Trouble falling or staying asleep, or sleeping too much
4. Feeling tired or having little energy
5. Poor appetite or overeating
6. Feeling bad about yourself – or that you are a failure or have let yourself or your family down
7. Trouble concentrating on things, such as reading the newspaper or watching television
8. Moving or speaking so slowly that other people could have noticed. Or the opposite – being so fidgety or restless that you have been moving around a lot more than usual
9. Thoughts that you would be better off dead, or of hurting yourself
10. If you checked off any problems, how difficult have these problems made it for you to do your work, take care of things at home, or get along with other people?
    - Not difficult at all
    - Somewhat difficult
    - Very difficult
    - Extremely difficult

*European Prospective Investigation into Cancer and Nutrition (EPIC) physical activity questionnaire [3] items*

1. We would like to know the type and amount of physical activity involved in your work. Please check what the best corresponds with your present occupation:

- Sedentary occupation: You spend most of your time sitting (such as in an office)
- Standing occupation: You spend most of your time standing and walking. However, your work does not require intense physical effort (e.g., shop assistant, hairdresser, guard, etc.)
- Manual work: This involves some physical effort including handling of heavy objects and use of tools (e.g., plumber, electrician, carpenter, etc.)
- Heavy manual work: This implies very vigorous physical activity including handling of very heavy objects (e.g., docker, miner, bricklayer, construction worker, etc.)

In a typical week during the past year, how many hours did you spend per week on each of the following activities:

1. Walking, including walking to work, shopping and leisure time
   1. In summer: […] hours
   2. In winter: […] hours
2. Cycling, including cycling to work, shopping and leisure time
   1. In summer: […] hours
   2. In winter: […] hours
3. Gardening
   1. In summer: […] hours
   2. In winter: […] hours
4. Do-it-yourself activities at home
   1. In summer: […] hours
   2. In winter: […] hours
5. Physical exercise such as fitness, aerobics, swimming, jogging, tennis, etc.
   1. In summer: […] hours
   2. In winter: […] hours
6. Housework, such as cleaning, washing, cooking, child care, etc.
   1. In summer: […] hours
   2. In winter: […] hours
7. In a typical week during the past year, did you engage in any of these activities vigorously enough to cause sweating or faster heartbeat?

- Yes
- No

1. [if yes] For how many hours per week in total did you perform vigorous activity?

[…] hours

1. In a typical week during the past year, how many flights of stairs did you climb per day?

[…] floors per day

*Cognitive reserve index questionnaire (CRIq) [4] items*

1. How many years of education (including postgraduate studies and any specialization) have you had? *Each school year successfully completed counts as 1 years, whereas each year that you were forced to repeat counts as 0.5 years.*

[…] years

1. How many years of vocational training have you had (with a clear training purpose and the presence of a teacher)? *Count 0.5 for every 6-month period of vocational training courses taken.*

[…] years

1. How many years have you carried out an occupation at one of the following levels? If you never worked, you can fill in everywhere.

- Low skilled manual work (farm work, gardener, housemaid, caregiver, waiter, driver, mechanic, plumber, call center operator, babysitter, etc.)

[…] years

- Skilled manual work (craftsman, cook, store clerk, tailor, representative, serviceman/servicewoman, hairdresser, clerical worker, nurse, etc.)

[…] years

- Skilled non manual work (business owner, white-collar employee, sales agent, priest or monk/nun, real estate agent, nursery school teacher, musician, etc.)

[…] years

- Professional occupation (Managing director of a small company, lawyer, qualified freelance professional, contractor, doctor, teacher, engineer, etc.)

[…] years

- Highly responsible or intellectual occupation (Managing director of a big company, senior manager, judge, university professor, surgeon, politician, etc.)

[…] years

1. Which of the following activities did you carry out **at least 3 times per week**, as an adult (from 18 years onwards)?

[For those activities selected]: please provide for how many years you did the activity as an adult. All paid activities or activities part of your education are not included here.

- Reading newspapers and magazines: […] years
- Domestic chores (cooking, washing, grocery shopping, ironing, etc.): […] years
- Driving (not biking): […] years
- Leisure activities (sports, hunting, dancing, chess, coin collecting, etc.): […] years
- Using new technologies (digital cameras, computer, Internet etc.): […] years

1. Which of the following activities did you carry out **at least 3 times per month**, as an adult (from 18 years onwards)?

[For those activities selected]: please provide for how many years you did the activity as an adult. All paid activities or activities part of your education are not included here.

- Social activities (political parties, recreational clubs, associations, etc.): […] years
- Cinema, theater: […] years
- Gardening, DIY, small-scale operations such as knitting, etc.: […] years
- Looking after grandchildren/nieces/nephews or elderly parents: […] years
- Voluntary work: […] years
- Artistic activities (music, singing, performance, painting, writing, etc.): […] years

1. Which of the following activities did you carry out **at least 3 times per year,** as an adult (from 18 years onwards)?

[For those activities selected]: please provide for how many years you did the activity as an adult. All paid activities or activities part of your education are not included here.

- Exhibitions, concerts, conferences: […] years
- Journeys lasting several days: […] years
- Reading books: […] years

1. Do you have children?

- Yes
- No

1. [If yes] How many children do you have?
2. Have you taken care of pets, as an adult?

- Never or rarely
- Often or always

1. [If selected “often or always”] How many years did you care for pets as an adult?
2. Have you managed your own current account as an adult?

- Never or rarely
- Often or always

1. [If selected “often or always”] How many years did you manage your own current account as an adult?

*Tobacco smoking items [5]*

1. Do you currently smoke tobacco?

- Daily
- Less than daily
- Never

1. [if selected “Daily”]: On average, how many of the following products do you currently smoke each day?

- […] Cigarettes per day
- […] Pipes full of tobacco per day
- […] Cigars or cigarillos per day
- […] Water pipe sessions per day

[If selected “Less than daily”]: On average, how many of the following products do you currently smoke each week?

- […] Cigarettes per week
- […] Pipes full of tobacco per week
- […] Cigars or cigarillos per week
- […] Water pipe sessions per week

1. How much do you weigh?

[…]kg

1. Wat is your height in centimeters?

[…] cm

Please state how much you agree or disagree with the following statements.

[Answer options:

- Agree strongly
- Agree
- Neither agree nor disagree
- Disagree
- Disagree strongly]

1. ‘There is nothing anyone can do to reduce their risks of getting dementia’
2. 'High blood pressure increases your chances of getting dementia'
3. ‘Smoking increases your chances of getting dementia’
4. ‘No or moderate alcohol use lowers your chances of getting dementia’
5. Regular physical activity lowers your chances of getting dementia’
6. ‘Depression increases the chances of getting dementia’
7. ‘Diabetes increases the chances of getting dementia’
8. ‘Being overweight increases the chances of getting dementia’
9. ‘A mentally active lifestyle lowers the chances of getting dementia’
10. ‘Heart disease increases the chances of getting dementia’
11. ‘Kidney disease increases the chances of getting dementia’
12. ‘High cholesterol increases the chances of getting dementia’
13. ‘Healthy diet lowers the chances of getting dementia’

Below multiple statements are shown. Can you indicate to what extent you agree or disagree with these statements? Every answer is good. Try to give the answer that fits your situation or beliefs best.

[Answer options:

- Agree strongly
- Agree
- Neither agree nor disagree
- Disagree
- Disagree strongly]

1. I have a healthy lifestyle
2. A healthy lifestyle is valuable
3. I want to live healthily
4. Starting to live a healthy/healthier lifestyle is tedious
5. Starting to live a healthy/healthier lifestyle is fun
6. I am sure I am capable of living a healthy lifestyle
7. I am motivated to live a healthy lifestyle
8. I feel confident I can live healthily

*Motivation to Change Behaviour for Dementia Risk Reduction Scale (MOCHAD-10) [6] items*

1. There is a strong possibility that I will develop dementia
2. When I think about dementia my heart beats faster
3. When I think about dementia I feel nauseous
4. The thought of dementia scares me
5. My feelings about myself would change if I develop dementia
6. Changing my lifestyle and health habits can help me reduce my chance of developing dementia
7. Having risk factor(s) for dementia makes me think I have to change my lifestyle and behavior
8. Knowing family member(s) with dementia makes me think I have to change my lifestyle and behavior
9. Learning more about dementia from the media makes me think I have to change my lifestyle and behavior
10. I am able to make differences that will change the risk of developing dementia
11. Can we contact you for participation in an interview at the end of this study? During this interview your opinion on the MijnBreincoach app and the GP consultation will be asked.

- Yes
- No

1. **Follow-up survey**

Please state how much you agree or disagree with the following statements.

[Answer options:

- Agree strongly
- Agree
- Neither agree nor disagree
- Disagree
- Disagree strongly]

1. ‘There is nothing anyone can do to reduce their risks of getting dementia’
2. 'High blood pressure increases your chances of getting dementia'
3. ‘Smoking increases your chances of getting dementia’
4. ‘No or moderate alcohol use lowers your chances of getting dementia’
5. Regular physical activity lowers your chances of getting dementia’
6. ‘Depression increases the chances of getting dementia’
7. ‘Diabetes increases the chances of getting dementia’
8. ‘Being overweight increases the chances of getting dementia’
9. ‘A mentally active lifestyle lowers the chances of getting dementia’
10. ‘Heart disease increases the chances of getting dementia’
11. ‘Kidney disease increases the chances of getting dementia’
12. ‘High cholesterol increases the chances of getting dementia’
13. ‘Healthy diet lowers the chances of getting dementia’
14. How often did you consume alcoholic beverages **in the last 3 months**?

- Never
- Maximum once per month
- 2-4 times per month
- 2-3 times per week
- 4 or more times per week

1. [If answer not "never"] On the days when you drank alcohol **in the past 3 months,** how many standard glasses did you typically consume? A standard glass is 25 cl of beer (4-5%), a small glass of wine, or a shot of strong liquor.

-
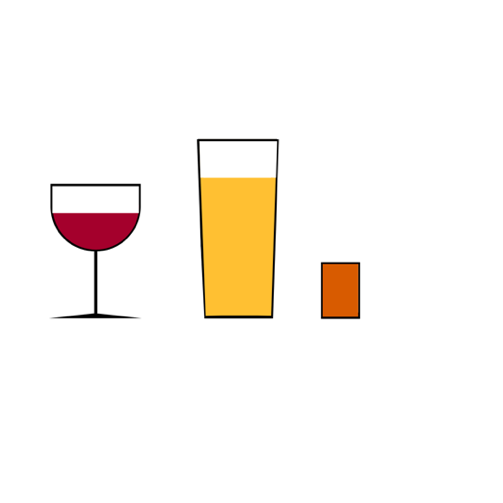
1-2 standard glasses
- 3-4 standard glasses
- 5-6 standard glasses
- 7-9 standard glasses
- 10 or more standard glasses

*Mediterranean diet adherence screener (MEDAS) items [1]*

1. Do you use olive oil as main culinary fat?

- Yes
- No

1. How much olive oil do you consume in a given day (including all oil used for frying, salads, out-of-house meals, etc.)?

- 0-1 tablespoon
- 2-3 tablespoons
- 4 or more tablespoons

1. How many serving spoons of vegetables (cooked and raw combined) do you eat per day?

- 0-3 serving spoons
- 4-7 serving spoons
- 7 or more serving spoons

1. How many fruit units (including natural fruit juices) do you consume per day? One unit of fruit is for example one average sized apple, one average-sized banana, two little mandarins, seven strawberries or one glass 100% natural fruit juice.

- 0 units
- 1 unit
- 2 units
- 3 or more units

1. How many servings of red meat or meat products (e.g., beef, pork, minced meat, sausage, game meats; but no poultry like chicken or turkey) do you consume per day? One serving is 100-150 g or the size of a deck of cards.

- Less than 1 serving
- More than 1 serving

1. How many servings of butter, margarine, or cream do you consume per day? One serving is about 1 tablespoon.

- Less than 1 serving
- More than 1 serving

1. How many sweet or carbonated beverages do you drink per day?

- Less than 1 glass
- 1 glass or more

1. How much wine do you drink per week?

- 0-6 glasses
- 7 or more glasses

1. How many servings of legumes (e.g., brown beans, white beans, peas, lentils) do you consume per week? 1 serving is 150 g or 3 tablespoons.

- 0-2 servings
- 3 or more servings

1. How many servings of fish or shellfish do you consume per week? 1 serving is 100-150 g or a medium-sized tin of salmon, tuna, or sardines.

- 0-2 servings
- 3 or more servings

1. How many times per week do you consume commercial sweets or pastries, such as candy, cakes, cookies, biscuits, or a sweet desert?

- 0-2 times
- 3 or more times

1. How many servings of nuts (including peanuts) do you consume per week? One serving is one handful.

- 0-2 servings
- 3 or more servings

1. Do you preferentially consume chicken, turkey, or rabbit instead of veal, pork, hamburger, or sausage?

- Yes
- No
- Not applicable

1. How many times per week do you consume dishes with sofrito (sauce made with tomato and onion, leek, or garlic, and olive oil?)

- 0-1 time
- 2 times or more

*Patient health questionnaire 9 (PHQ-9) items [2]*

Over the last 2 weeks, how often have you been bothered by any of the following problems?

[Answer options:

- Not at all
- Several days
- More than half of the days
- Nearly every day]

1. Little interest or pleasure in doing things
2. Feeling down, depressed, or hopeless
3. Trouble falling or staying asleep, or sleeping too much
4. Feeling tired or having little energy
5. Poor appetite or overeating
6. Feeling bad about yourself – or that you are a failure or have let yourself or your family down
7. Trouble concentrating on things, such as reading the newspaper or watching television
8. Moving or speaking so slowly that other people could have noticed. Or the opposite – being so fidgety or restless that you have been moving around a lot more than usual
9. Thoughts that you would be better off dead, or of hurting yourself
10. If you checked off any problems, how difficult have these problems made it for you to do your work, take care of things at home, or get along with other people?
    - Not difficult at all
    - Somewhat difficult
    - Very difficult
    - Extremely difficult

*European Prospective Investigation into Cancer and Nutrition (EPIC) physical activity questionnaire [3] items*

1. Did you change occupation in the last 3 months?

- Yes
- No

1. [If yes] We would like to know the type and amount of physical activity involved in your work. Please check what the best corresponds with your present occupation:

- Sedentary occupation: You spend most of your time sitting (such as in an office)
- Standing occupation: You spend most of your time standing and walking. However, your work does not require intense physical effort (e.g., shop assistant, hairdresser, guard, etc.)
- Manual work: This involves some physical effort including handling of heavy objects and use of tools (e.g., plumber, electrician, carpenter, etc.)
- Heavy manual work: This implies very vigorous physical activity including handling of very heavy objects (e.g., docker, miner, bricklayer, construction worker, etc.)

In a typical week during **the last 3 months**, how many hours did you spend per week on each of the following activities:

1. Walking, including walking to work, shopping and leisure time
   - In summer: […] hours
   - In winter: […] hours
2. Cycling, including cycling to work, shopping and leisure time
   - In summer: […] hours
   - In winter: […] hours
3. Gardening
   - In summer: […] hours
   - In winter: […] hours
4. Do-it-yourself activities at home
   - In summer: […] hours
   - In winter: […] hours
5. Physical exercise such as fitness, aerobics, swimming, jogging, tennis, etc.
   - In summer: […] hours
   - In winter: […] hours
6. Housework, such as cleaning, washing, cooking, child care, etc.

- In summer: […] hours
- In winter: […] hours

1. In a typical week during the **last 3 months**, did you engage in any of these activities vigorously enough to cause sweating or faster heartbeat?

- Yes
- No

1. [if yes] For how many hours per week in total did you perform vigorous activity?

[…] hours

1. In a typical week during **the past 3 months**, how many flights of stairs did you climb per day?

[…] floors per day

1. Have you done any of the following activities more or less frequently during the last 3 months compared to before?

[Answer options:

- Much more often
- A bit more often
- Same frequency
- A bit less often
- Much less often]
  - Reading newspapers and magazines
  - Domestic chores (cooking, washing, grocery shopping, ironing, etc.)
  - Driving (not biking)
  - Leisure activities (sports, hunting, dancing, chess, coin collecting, etc.)
  - Using new technologies (digital cameras, computer, Internet etc.)
  - Social activities (political parties, recreational clubs, associations, etc.)
  - Cinema, theater
  - Gardening, DIY, small-scale operations such as knitting, etc.
  - Looking after grandchildren/nieces/nephews or elderly parents
  - Voluntary work
  - Artistic activities (music, singing, performance, painting, writing, etc.)
  - Exhibitions, concerts, conferences
  - Journeys lasting several days
  - Reading books

*Tobacco smoking items [5]*

1. Do you currently smoke tobacco?

- Daily
- Less than daily
- Never

1. [if selected “Daily”]: On average, how many of the following products do you currently smoke each day?

- […] Cigarettes per day
- […] Pipes full of tobacco per day
- […] Cigars or cigarillos per day
- […] Water pipe sessions per day

[If selected “Less than daily”]: On average, how many of the following products do you currently smoke each week?

- […] Cigarettes per week
- […] Pipes full of tobacco per week
- […] Cigars or cigarillos per week
- […] Water pipe sessions per week

1. How much do you weigh?

[…]kg

Below multiple statements are shown. Can you indicate to what extent you agree or disagree with these statements? Every answer is good. Try to give the answer that fits your situation or beliefs best.

[Answer options:

- Agree strongly
- Agree
- Neither agree nor disagree
- Disagree
- Disagree strongly]

1. I have a healthy lifestyle
2. A healthy lifestyle is valuable
3. I want to live healthily
4. Starting to live a healthy/healthier lifestyle is tedious
5. Starting to live a healthy/healthier lifestyle is fun
6. I am sure I am capable of living a healthy lifestyle
7. I am motivated to live a healthy lifestyle
8. I feel confident I can live healthily

*Motivation to Change Behaviour for Dementia Risk Reduction Scale (MOCHAD-10) [6] items*

1. There is a strong possibility that I will develop dementia
2. When I think about dementia my heart beats faster
3. When I think about dementia I feel nauseous
4. The thought of dementia scares me
5. My feelings about myself would change if I develop dementia
6. Changing my lifestyle and health habits can help me reduce my chance of developing dementia
7. Having risk factor(s) for dementia makes me think I have to change my lifestyle and behavior
8. Knowing family member(s) with dementia makes me think I have to change my lifestyle and behavior
9. Learning more about dementia from the media makes me think I have to change my lifestyle and behavior
10. I am able to make differences that will change the risk of developing dementia

Please state how much you agree or disagree with the following statements.

[Answer options:

- Agree strongly
- Agree
- Neither agree nor disagree
- Disagree
- Disagree strongly]

1. Discussing my health profile gave me more insight in the relationship between my lifestyle and health.
2. Discussing my health profile with my GP was useful and interesting.
3. Seeing my health profile was confronting.
4. Discussing my health profile motivated me to make lifestyle improvements
5. Did you install the MijnBreincoach app?

- Yes
- No

1. [If yes]: Did you use the MijnBreincoach app?

- Yes
- No

[If no]: Why did you not install the app?

Open answer

[The next questions are only asked if the participant used the MijnBreincoach app]

Please state how much you agree or disagree with the following statements.

[Answer options:

- Agree strongly
- Agree
- Neither agree nor disagree
- Disagree
- Disagree strongly]

1. The page that showed my room for improvement in the MijnBreincoach app was clear.
2. I find the presentation of the messages through “nuts” appealing.
3. The content of the daily messages (cracked nuts) is easy to read.
4. The tips and advice in the app fit my personal situation well.
5. I think the quiz questions make a valuable contribution to the MijnBreincoach app.
6. The images used in the MijnBreincoach app are closely aligned with the content of the messages.
7. I was able to make use of the tips and advice from the MijnBreincoach app.
8. I regularly visited the website of the organization referred to in the messages (cracked nuts).
9. I find the challenges to be a meaningful contribution to the MijnBreincoach app.
10. It was clear to me how I could collect points in the app.
11. Collecting points motivated me to continue using the MijnBreincoach app.
12. I generally find the MijnBreincoach app easy to use.
13. I enjoyed using the MijnBreincoach app.
14. Using the MijnBreincoach app caused irritation for me.
15. I found it convenient that I could reread the cracked nuts.
16. With the help of the MijnBreincoach app, I worked on improving my lifestyle in a positive way.
17. Through the use of the MijnBreincoach app, I gained more knowledge about the concept of brain health.
18. The MijnBreincoach app has given me a better understanding of organizations or entities that can assist me in achieving a healthier lifestyle (examples of several Dutch organizations).
19. The MijnBreincoach app has helped me make changes to my lifestyle.
20. I will continue to use the MijnBreincoach app.
21. The MijnBreincoach app has made me reflect on my own brain health.
22. Would you recommend the app?

- No
- Maybe
- Yes

1. How much would you rate the MijnBreincoach app on 10?

Number between 0 and 10

Please state how much you agree or disagree with the following statements.

[Answer options:

- Agree strongly
- Agree
- Neither agree nor disagree
- Disagree
- Disagree strongly]

1. I have positively worked on my lifestyle in the past 3 months.
2. I have achieved my set lifestyle goal(s).
3. Were there factors that made it more difficult for you to achieve your lifestyle goals?

You can select more than 1 option.

- Insufficient time
- My financial situation
- Limited opportunities in my environment (facilities or infrastructure, e.g., lack of or poor bike paths if you want to exercise more)
- No one in my surroundings is doing this
- I was afraid that I would feel less connected to my friends, colleagues, and/or family
- I was afraid that I would have less fun
- I don't think I need to change my lifestyle (insufficient motivation)
- I quickly realized that it wouldn't be possible to achieve my lifestyle goals
- Insufficient skills (e.g., cooking)
- I didn't see immediate benefits from my healthy behavior
- None of the above
- Other: open response

1. Were there factors that made it easier for you to work on your lifestyle goals?

You can select more than 1 option.

- My friends and/or family supported me
- I felt good taking responsibility for my health
- I enjoy living a healthy lifestyle
- I saw quick results
- None of the above
- Other: open response

1. The MijnBreincoach app has made it easier for me to lead a healthier lifestyle.

- Strongly agree
- Agree
- Neither agree nor disagree
- Disagree
- Strongly disagree

1. Have you used any other health apps in the past 3 months? These are apps you might use to improve or monitor your health, such as sleep monitors, apps to help you quit smoking, calorie counters (e.g., MyFitnessPal), fitness apps (e.g., Freeletics), step counters (e.g., Google Fit).

- Yes
- No

1. [If yes]: Which apps?

Open answer

1. Did you see a dietician or lifestyle coach during the last 3 months?

- Yes
- No

**REFERENCES**

[1] Martinez-Gonzalez MA, Garcia-Arellano A, Toledo E, Salas-Salvado J, Buil-Cosiales P, Corella D, Covas MI, Schroder H, Aros F, Gomez-Gracia E, Fiol M, Ruiz-Gutierrez V, Lapetra J, Lamuela-Raventos RM, Serra-Majem L, Pinto X, Munoz MA, Warnberg J, Ros E, Estruch R, Investigators PS (2012) A 14-item Mediterranean diet assessment tool and obesity indexes among high-risk subjects: the PREDIMED trial. *PLoS One* **7**, e43134.

[2] Kroenke K, Spitzer RL, Williams JB (2001) The PHQ-9: validity of a brief depression severity measure. *J Gen Intern Med* **16**, 606-613.

[3] Cust AE, Smith BJ, Chau J, van der Ploeg HP, Friedenreich CM, Armstrong BK, Bauman A (2008) Validity and repeatability of the EPIC physical activity questionnaire: a validation study using accelerometers as an objective measure. *Int J Behav Nutr Phys Act* **5**, 33.

[4] Nucci M, Mapelli D, Mondini S (2012) Cognitive Reserve Index questionnaire (CRIq): a new instrument for measuring cognitive reserve. *Aging Clin Exp Res* **24**, 218-226.

[5] Global Adult Tobacco Survey Collaborative Group (2011), Atlanta, GA.

[6] Oliveira D, Aubeeluck A, Stupple E, Kim S, Orrell M (2019) Factor and reliability analysis of a brief scale to measure motivation to change lifestyle for dementia risk reduction in the UK: the MOCHAD-10. *Health Qual Life Outcomes* **17**, 75.
