# Supplementary material for: Multidomain Dementia Risk Reduction in Primary Care is Feasible: A Proof-of-concept study
Source: J Alzheimers Dis. 2024 Jun 11;99(4):1455–71. doi: 10.3233/JAD-240229 (PMC11191460; doi:10.3233/JAD-240229)
Supplement: Supplementary Material 3 [file jad-99-jad240229-s003.docx]

# **Supplementary Table 1. Interview guide - PRIMA-Brain participants**

| Time | **Introduction**  *Aim: Introduce yourself and explain why we conduct these interviews.*  *Give the interviewee the chance to ask questions.* |
| --- | --- |
| 5 min | Thank you for attending and participation in the study.  Introduce yourself.  INTERVIEWER:  Let me introduce myself first. My name is […]. I work as a researcher at the Alzheimer Center Limburg and Maastricht University. Today, I will interview you about your participation in the study, the consultation in the general practitioner's office about your lifestyle, the use of the MyBraincoach app, and making lifestyle changes.  Why are we conducting this interview?  Today, there is little awareness of brain health and what individuals can do themselves to improve their brain health. This is something we want to address. A potentially low-level intervention that can be implemented on a large scale could be, to make the topic of brain health a regular topic discussion in primary care. But we want get a thorough idea of whether this is feasible and how people would experience this.  🡪 Central question: What is important if we would implement this intervention in the general practice? We are curious for your sincere, honest, critical opinion, and experiences. Feel free to share everything with us; we are very grateful for your input.  Audio-recording of the interview  We would like to record this interview. The recording will be treated confidentially and deleted within 3 months after transcription. Do you agree with this? |
|  | **Part 1: General experience and value of the intervention** |
| 3 min | How have you experienced your participation?  Why good/bad?  Any problems or issues? |
|  | **Part 2: Reasons for participation (what was the participant’s motivation?)** |
| 2 min | Why did you want to participate in this study? |
|  | **Part 3: the MyBraincoach app** |
| 15 min | After your visit to the general practitioner's office, you installed the MyBraincoach app on your phone. In doing so, you could choose a theme and “crack a walnut” daily, which provided information about that theme. *Show screenshots.*  How often did you use the app? What was your overall impression of the app?  What did you like about the app? What did you dislike about it?  Is there anything that you would change about the app?  What would you find enjoyable?  What is missing for you?  Did you find the app relevant for you?  Would you recommend the MyBraincoach app to others?  Did you learn anything from the app?  What did you think of the content of the “nuts”?  Was the app user-friendly?   - Was there anything in the app that was difficult to use? - Readability?   What did you think of the layout?  Do you feel that the app can be supportive or helpful for a healthy lifestyle?   - Which specific parts of the app, if you recall. - How else could the app help or provide support? New elements that could be added? |
|  | **Part 4: the consultation at the general practice** |
| 10 min | With whom did you have this conversation (if you still remember)?  How long did this conversation approximately last?  How did you experience this consultation?  What did you like about it? What did you dislike?  Did you learn anything from this consultation?  Would you have liked something to have been done differently?   - What was missing for you? - What would you do or like to be done differently? |
|  | **Part 5: Behavior change** |
| 10 min | Did you set a lifestyle goal during the consultation in the general practitioner's office?  Was there something you personally wanted to change about your lifestyle?  How actively did you implement changes into your lifestyle based on the information from the consultation and the app?  Have you made any changes to your lifestyle, no matter how small?  What makes it difficult for you to implement changes in your lifestyle?  (If nothing comes to mind: for example, work-family balance, children, stress, money, time, not important, etc.)  What do you think would make it easier for you to work on your lifestyle goal?  What would you need?  Can you think of anything that would help you (or people in general) to live a healthier life? |
|  | **Part 6: Recommendations** |
| 5 min | Do you have any other suggestions, advice, or comments for us?  Are there elements you would like to add to the intervention, or leave out? |
|  | **End** |
| 2 min | Thank you! These were all the questions that I had for you today.  Is there anything else that you want to share with me?  Thank you for your participation. |

# **Supplementary Table 2. Interview guide – primary care providers**

| Tijd | **Introduction**  *Aim: Introduce yourself and explain why we conduct these interviews.*  *Give the interviewee the chance to ask questions.* |
| --- | --- |
| 5 min | Thank you for attending and participation in the study.  Introduce yourself.  INTERVIEWER:  Let me introduce myself first. My name is […]. I work as a researcher at the Alzheimer Center Limburg and Maastricht University. Today, I will interview you about your participation in the study, the conversation in the general practitioner's office about your lifestyle, the use of the MyBraincoach app, and making lifestyle changes.  Why are we conducting this interview?  Today, there is little awareness of brain health and what individuals can do themselves to improve their brain health. This is something we want to address. A potentially low-level intervention that can be implemented on a large scale could be, to make the topic of brain health a regular topic discussion in primary care. But we want get a thorough idea of whether this is feasible and how people would experience this.  🡪 Central question: What is important if we would implement this intervention in the general practice? We are curious for your sincere, honest, critical opinion, and experiences. Feel free to share everything with us; we are very grateful for your input.  Audio-recording of the interview  We would like to record this interview. The recording will be treated confidentially and deleted within 3 months after transcription. Do you agree with this? |
|  | **Part 1: General experience and value of the intervention in general** |
| 5 min | How did you experience the participation?  Has participation in the study led to specific questions from participating patients?  Did you experience any problems?  Have you talked about brain health or dementia risk reduction in the past as primary care provider? |
|  | **Part 2: The consultation with the patient/participant** |
| 15 min | How did you experience these consultations?  Would it be feasible for you to make “brain health and dementia” a regular topic at the general practice, next to the already existing cardiovascular risk management?  Would this be desirable according to you?  Is this of added value to the current health system?  Do you think there would there be a demand for this?  Are there barriers before we could implement this? What would be required?  Are there things that could facilitate this implementation or help primary care professionals towards this?  Was the provided brain health profile usable and/or helpful?  Do you think you will continue to discuss brain health in the future?  Why? Why not? |
|  | **Part 3: MyBraincoach app** |
| 5-10 min | What do you think about offering an app about improving brain health, to patients in the general practice?  How would you do this specifically? For example, mention it verbally, providing a leaflet,…? Do you think you would do this personally (refer to the app)?  What do you think would be necessary or helpful to get patients to use the app? |
|  | **Part 4: General recommendations** |
| 5 min | Do you have any other recommendations for us?  Are there things you would alter about this intervention? |
|  | **End** |
| 5 min | Thank you! These were all the questions that I had for you today.  Is there anything else that you want to share with me?  Thank you for your participation. |
