# Supplementary material for: Multidomain Dementia Risk Reduction in Primary Care is Feasible: A Proof-of-concept study
Source: J Alzheimers Dis. 2024 Jun 11;99(4):1455–71. doi: 10.3233/JAD-240229 (PMC11191460; doi:10.3233/JAD-240229)
Supplement: Supplementary Material 4 [file jad-99-jad240229-s004.docx]

**Calculation of the LIfestyle for BRAin health (LIBRA) score**

The LIfestyle for BRAin health (LIBRA) score is calculated by summing the weights of all risk and protective factors that are present within an individual (see Table 1).

**Table 1**: Risk and protective factors included in the LIfestyle for BRAin health (LIBRA) score and their associated weights

| **LIfestyle for BRAin health (LIBRA) factors** | **Weight** |
| --- | --- |
| Physical inactivity | +1.1 |
| Current smoker | +1.5 |
| Obesity | +1.6 |
| Hypertension | +1.6 |
| Dyslipidemia | +1.4 |
| Diabetes | +1.3 |
| Depression | +2.1 |
| Chronic kidney disease | +1.1 |
| Coronary heart disease | +1.0 |
| Low-to-moderate alcohol consumption | -1.0 |
| High cognitive activity | -3.2 |
| Healthy diet | -1.7 |
